# Hardness changing tactile displays for simulating the feel of organic tissues

**DOI:** 10.3389/frobt.2024.1404543

**Published:** 2024-08-20

**Authors:** Joshua Brown, Fernando Bello

**Affiliations:** Simulation and Modelling in Medicine and Surgery (SiMMS) Lab, Department of Surgery and Cancer, Imperial College London, London, United Kingdom

**Keywords:** haptics, soft robotics, tactile displays, particle jamming, medical robotics, medical simulation

## Abstract

Physical interaction with patients, for example conducted as part of a diagnostic examination or surgical procedure, provides clinicians with a wealth of information about their condition. Simulating this interaction is of great interest to researchers in both haptics and medical education, and the development of softness changing tactile interfaces is important in recreating the feel of different soft tissues. This paper presents designs for a variety of novel electromechanical and electromagnetic mechanisms for controlling particle jamming-based, hardness changing tactile displays, intended to allow medical trainees to experience these physical interactions in a range of simulation settings such as clinical skills teaching laboratories. Each design is then subjected to a battery of mechanical tests to evaluate its effectiveness compared to the state of the art, as well as their suitability for simulating the physical hardness of different types of soft tissues, previously characterised in established literature. These results demonstrate that all of the technologies presented are able to exhibit a measurable hardness change, with Shore hardness values between 3A and 57A achieved by the most effective constriction-based device. The electromechanical devices based on constriction and compression, and the state-of-the-art pneumatic device, were able to achieve hardness changes within a range that is useful for replicating the softness of organic tissue. The electromechanical and electromagnetic devices were also found to effect their full range of hardness change in less than a second, compared to several seconds for the state-of-the-art. These results show that the performance of softness changing tactile displays can be improved with the electromechanical actuation techniques proposed in this paper, and that such displays are able to replicate the physical characteristics of soft tissues and may therefore be of benefit in medical training and simulation scenarios.

## 1 Introduction

Physical examination is a vital diagnostic tool in medicine. A trained and experienced clinician can determine a wealth of information about the nature and severity of a patient’s condition through the texture of their skin, the hardness and size of a lump or organ, the strength and character of their pulse, and even the temperature of a rash or limb. As critically important as these physical, tactile signals are, there remain a number of questions about the best way to demonstrate them to medical students as they learn to examine patients for the first time. A safe space, away from the pressures of real patients or the pressures of a hospital ward is essential, as is useful, relevant and actionable feedback regarding their performance. These demands strongly advocate for a simulation approach to training physical examination. What is missing is the ability to accurately render haptic cues as they would present on an unwell patient.

Hardness can indicate a number of different clinical pathologies. A hardened lymph node might be cancerous and require urgent investigation. Firmness in the calf can indicate abnormal blood flow and require medication to treat. A hard lump could be a fluid filled cyst and require surgery to remove. Existing research has measured the mechanical hardness (expressed in Shore units on either the 00, A, C or D scales) of a number of different soft tissues. Work reported in Estermann et al. (2020) found that porcine and bovine liver samples measure around 30A and 26A respectively, whilst Baumgart et al. (2010) has found that the hardness of a cancerous prostate can range from 23 to 31A. Additionally, Tonna et al. (2024) found that plantar skin has a shore hardness in the range of 15 and 30A depending on the region of the foot being measured, though a study presented in Piaggesi et al. (1999) reported much higher values up to 52A in patients with diabetes. Whilst durometry has been proposed as a method of diagnosing various skin conditions by Kissin et al. (2006) and Aghassi et al. (1995), the relatively low thickness of the skin means that the bulk tissue behind it has a more pronounced effect on perceptible hardness. This effect is discussed at length in Chatzistergos et al. (2022), making apparent hardness a highly relevant metric for simulating pathologies such as lumps and tumors in haptic training models.

Unfortunately, methods of dynamically simulating hardness in the classroom generally rely on expensive force-feedback robots, or noisy, heavy soft robotic actuators. A force feedback robot, programmed with a suitable force-displacement curve as demonstrated in Ullrich and Kuhlen (2012), can be a useful training aid for palpation. Hardness changing soft robots, such as the shape and hardness changing table described in Stanley et al. (2013), can produce even more realistic simulations of soft tissues and anatomical features, but are not yet ready for deployment in the real world.

Particle jamming, a physical phenomenon first described scientifically in Biroli (2007), refers to the tendency of granular fluids to become hard or stiff when their individual constituent particles are compacted and forced against each other. This compaction is achieved by evacuating the air from a silicone pouch or sack that is filled with the particles, causing the airtight outer surface to contract inwards and tightly squeeze the particles. This effect has a number of applications in engineering and robotics, ranging from universal, shape independent robotic grippers described in Brown et al. (2010), to hardness and shape changing haptic and HCI devices, first explored and discussed in Follmer et al. (2012).

Since this original work, particle jamming has been used in a number of ways to create a wide range of different types of haptic devices. Stiffness changing pouches can be attached to gloves, as has been demonstrated using particles by Zubrycki and Granosik (2017), and layers of fabric by Simon et al. (2014) to restrict the movement of fingers when grasping and manipulating objects, or clothing, to hold other joints in place in response to events in a virtual reality game Al Maimani and Roudaut (2017). Multimodal tactile devices have also been created using particle jamming, with particle jamming and vibration being combined in both large format tactile experiences explored in Kurihara et al. (2014), and interactive, desktop scale interfaces such as the joystick and touchpad discussed in Brown and Farkhatdinov (2022) and Brown and Farkhatdinov (2021).

Particle jamming has already been demonstrated as an effective basis for building soft medical simulators. Projects described in Li et al. (2014) and Stanley and Okamura (2017) have combined particle jamming pouches with pneumatic shape changing systems to allow the creation of lumps and areas of abnormal hardness on a virtual patient. This work was subsequently extended in Stanley et al. (2014) to create a hardness and shape changing encountered type tactile display over which 3D graphics of anatomy could be overlaid to support teaching and simulation.

Despite proven effectiveness in creating convincing haptic experiences and simulations, particle jamming is not generally considered a viable technology for use in consumer or otherwise mass market devices and systems. This is due to the considerable size and weight of the pneumatic apparatus that is required to drive such systems. An open challenge in haptics and soft robotics is therefore the design of an effective, affordable, quiet and lightweight particle jamming system that can be operated and controlled without bulky, expensive pneumatic hardware.

## 2 Actuation techniques

Several approaches to actuating a particle jamming-based tactile device were devised, built and tested. These are described below. Two of the devices are mechanical and driven entirely by electric motors Brown and Bello (2024). These are compared to a further magnet-based device and a conventional, vacuum-operated tactile display.

### 2.1 Constriction

The first non-pneumatic approach that will be explored in this work is constriction. In this design, the traditional silicone container is replaced by an inelastic fabric container. This is secured at one end (the top) to a rigid frame, whilst the rest of the container is allowed to hang freely and filled with the particle fluid. A high-torque motor attached to the other end of the pouch can then twist it around the fluid and, because the container is not elastic and will not stretch to relieve its tension, exert a force on the fluid from all sides. This principle bears some resemblance to twisted string actuation, discussed at length in Gaponov et al. (2013) and Würtz et al. (2010) applied to haptic interfaces in Skvortsova et al. (2022) and Hosseini et al. (2018), as well as fiber jamming, described in more detail in Brancadoro et al. (2020).

**FIGURE 1:**
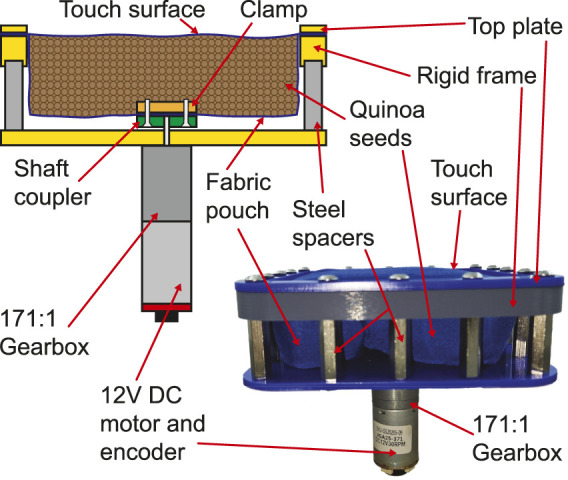
Schematic view of a hardness changing tactile display based on constriction of a particle fluid, and a photograph of the working prototype.

A prototype tactile display using this principle was designed and built. In this device, a 100 mm × 100 mm × 30 mm pouch made from thin fabric was produced. This was then suspended from a rigid frame made from 3D printed and laser cut plastic plates and steel hex spacers. A 12V DC motor with a 171:1 high-torque gearbox was then secured under the pouch and it is shaft clamped to the bottom of the fabric pouch such that turning the motor would twist the bottom of the pouch relative to the top. The pouch was filled with Quinoa seeds (approx 1.5 mm dia) to act as the particle fluid. A second fabric sheet was clamped to the top with a plastic plate and screws. The schematic view of this device and a photograph of the prototype are shown in Figure 1.

### 2.2 Compression

The compression approach was informed by the observation in Brown and Farkhatdinov (2020) that a human user pressing down on a particle jamming-based haptic device causes the particles to jam together. A viable particle jamming-based tactile display can therefore be created by using a linear piston type mechanism to compress a particle fluid within a container that is, again, inelastic. In this way, extending the piston into the container volume will force the particles together causing them to jam, whilst retracting the piston will release the inter-particle forces and relax the fluid.

**FIGURE 2:**
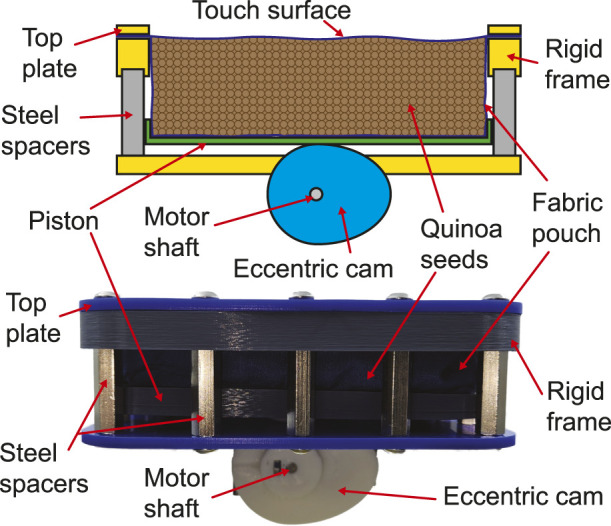
Schematic view of a hardness changing tactile display based on compression of a particle fluid, and a photograph of the working prototype.

To demonstrate this principle, a 100 mm × 100 mm × 30 mm pouch made from the same inelastic fabric was again filled with quinoa seeds. A rigid plastic plate was then positioned under it, resting on an eccentric cam, in turn connected to a highly geared DC motor. Turning the motor therefore has the effect of lifting the plate up into the particle fluid, compressing it against the top of the bag. Reversing the cam then allows the mass of the particles, under gravity, to push the plate back down, releasing the forces and softening the interface. This prototype is shown in Figure 2.

### 2.3 Magnetism

One of the engineering benefits of particle jamming is that the specification of the particles can be set to meet the specific requirements of the device it is being used to create. In other words, as long as they are solid and round, it does not matter what they are made from. Using ferromagnetic particles (for example, made from iron or steel) raises the possibility of a magnet, either embedded in the fluid or positioned under it, being used to attract particles and force them together towards the magnet.

**FIGURE 3:**
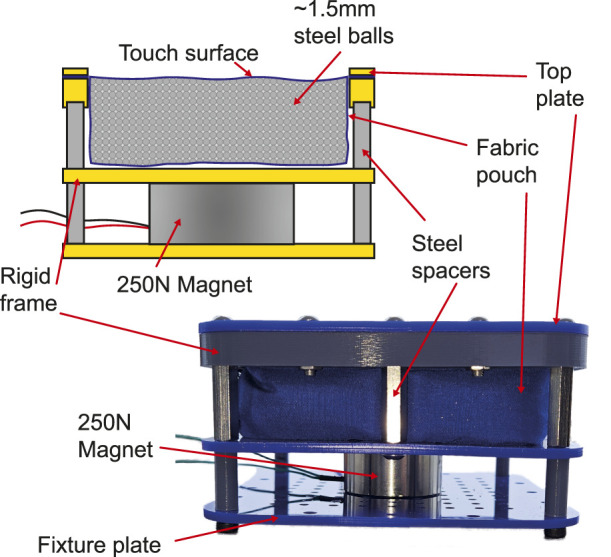
Schematic view of a hardness changing tactile display based on using a magnet to attract and compress ferromagnetic particles, and a photograph of the working prototype.

To test this approach, a 12V, 250N electromagnet was secured to a frame underneath a 100 mm × 100 mm × 30 mm fabric pouch filled with AISI 1010 Carbon Steel ball bearings. These were specified with a 1.5 mm diameter such that their behaviour could be compared with the quinoa seeds used in the other interfaces. The pouch was suspended from the same rigid plastic and steel frame used previously. A photograph and schematic view of this device are shown in Figure 3.

### 2.4 Vacuum

Actuation using a vacuum is the prevailing approach for actuating particle jamming-based actuators, both for haptic devices and other soft robots. This mechanism works by squeezing a soft, airtight pouch around the particle fluid when a region of low pressure is created inside it. This squeezing forces the particles together, causing the fluid as a whole to harden. Adjusting the strength of the negative pressure adjusts the hardness of the fluid.

**FIGURE 4:**
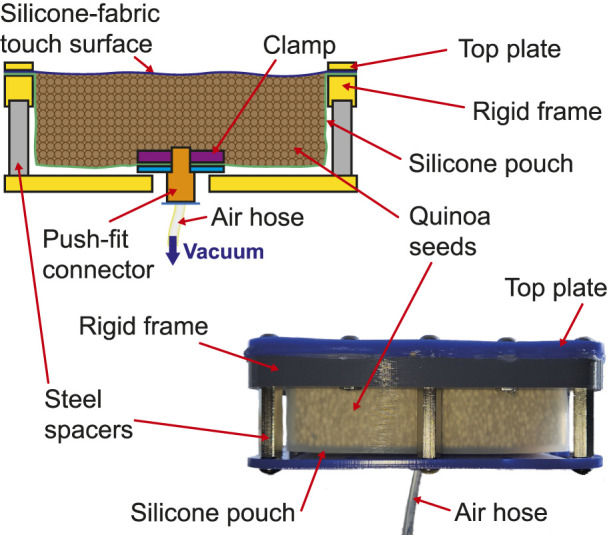
Schematic view of a hardness changing tactile display based on constriction, and a photograph of the working prototype.

In order to enable the above described prototypes to be benchmarked against the current state-of-the-art, a vacuum operated particle jamming-based tactile display was created. This has identical dimensions (100 mm × 100 mm × 30 mm) as the prototype devices presented. Air-tightness was maintained by using a 2 mm thick silicone (Smooth-On Ecoflex 00-20) pouch. The effect of the soft silicone layer on the measurable hardness of the top surface was reduced by soaking the thin fabric in the same silicone to make it airtight, but still thin and strong enough to properly transfer the hardness change caused by the jamming. A 4 mm pneumatic push-fit connector was screwed between two plastic plates in the bottom of the pouch and covered with fabric to stop the particle fluid being sucked out of the device. This device, and it is schematic cross-section, is shown in Figure 4.

## 3 Experimental characterisation

Several mechanical tests were performed on the prototype tactile displays described above to evaluate the effectiveness of each approach for simulating the tactile hardness of soft tissues.

### 3.1 Shore hardness

Hardness refers to a material’s surface deformation when a load is applied and is usually measured on the Shore 00, A, C and D scales using a durometer instrument. Belyaev et al. (2010) has found that Shore hardness is an effective proxy for the perceived feel of soft tissue by surgeons during palpation. Since hardness is the key haptic effect presented by these actuators and is known to be a key clinical indicator when palpating tissues during examination or surgery, it is important to quantify both the expected range of hardness change of the different technologies, and the ease and repeatability with which this can be controlled to mimic the feel of a specific organ/condition.

**FIGURE 5:**
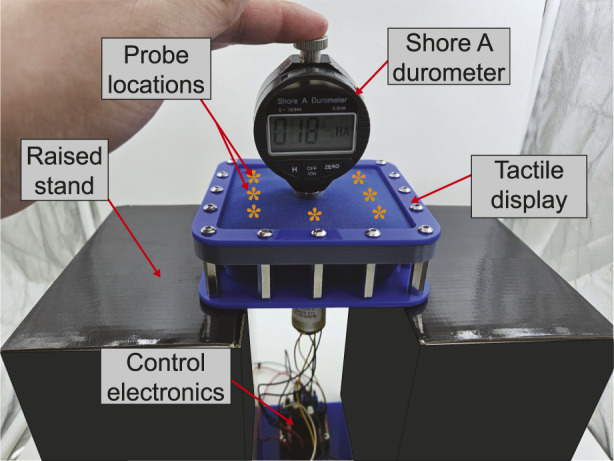
Shore hardness testing setup pictured with the constriction prototype. Other devices were tested in the same way, except for the magnetic prototype being driven by a benchtop power supply and the vacuum device being driven by a vacuum pump and regulator.

Hardness can be measured using a handheld durometer, which consists of a spring loaded steel needle protruding from a flat plate. When the plate is pressed flat into the material, the displacement of the needle is used to calculate the localised hardness of the surface. To measure the hardness of the prototype tactile displays, a durometer set to measure hardness on the Shore A scale^1^ was used to probe nine locations on the touch surface - the centre as well as eight points in a 25 mm spaced grid around it, as shown in Figure 5. These locations were first probed with the devices in their soft states (no rotation/compression, magnet and vacuum switched off) before being repeated as each device was stepped closer to its maximum theoretical hardness, defined as:

• Constrictive pouch rotated to 180°, in 10°increments

• Piston extended by 10 mm, in approx 0.6 mm increments

• Magnet powered by 12 V, in 1 V increments

• −50 kPa vacuum pressure, in 5 kPa increments

This procedure was repeated three times for each device. After each device reached it is maximum rotation/extension/power/vacuum in a test, it was reset to zero and shaken to relieve any residual jamming effects before being tested again. The results for each trial/point/hardness step were averaged and used to calculate minimum-maximum achievable hardness (based on the centre of each display), as well as hardness uniformity across the surface (based on the average difference between the centre and each surrounding point). The experiment setup and location of each sampling point are shown in 5.

An additional test was conducted on the vacuum operated tactile display to investigate the effect of stronger negative pressure. Here, the vacuum pump was connected directly to the tactile display and switched on, reaching a pressure of approximately −100 kPa. The surface hardness was then recorded using the procedure above.

### 3.2 Response time

Response time is also a vitally important performance indicator for interactive HCI devices. An interface that responds to command input too slowly will, at best, not produce realistic sensations and, at worst, cause visuo-haptic incongruence that can lead to simulator sickness, as demonstrated in Draper et al. (2001). This is vital in medical training where not only is the hardness of a pathology important, but also it is size and shape - features that both require responsive haptic output to render correctly during exploratory palpation.

Response time was measured differently for each interface. The electromechanical designs - constriction and compression, were modified such that their motion controllers moved smoothly (but as fast as possible) from their minimum to their maximum output and reported the time taken. The electromagnet was connected to a power source and an oscilloscope used to measure the voltage gain across the coil, identifying the time taken for the magnet to reach maximum strength. The vacuum actuated device’s regulator was switched quickly from it is minimum (atmospheric) to its maximum response and a high speed camera (240fps) used to record the change. This was then played back frame by frame to determine the time taken for the silicone pouch to conform to a solid shape around the particles. Again, each of these tests was repeated three times and the results averaged.

## 4 Results

The results of the above tests are analysed and presented below.

### 4.1 Range of hardness response

The minimum hardness measured in each device fell between 3A and 5A on the Shore scale. These numbers represent very little difference. Whilst this is to be expected given the devices’ identical dimensions and particle sizes, it is useful to confirm that the additional weight of the steel particles in the magnetic version of the interface and material change in the vacuum powered device had a negligible, if any, effect on the hardness of each surface.

The maximum hardness achieved by the four devices was highly variable. The constriction prototype achieved the hardest surface under controlled operation with a peak hardness of 57A. The compression-based device achieved a lower value of 42.5A with the vacuum powered device reaching a peak of 36A in the centre of the touch surface. The magnetic device achieved a hardness of 10A in the centre, but did record higher values of up to 15A around the edges. The stronger, unregulated vacuum was able to produce a 68A surface, which was the hardest recorded during this study and represents the current state-of-the-art for particle jamming based tactile displays.

All four devices demonstrated good controllability and repeatability. Each required some fraction of its initial response to take up slack in the fluid or the pouch, then demonstrated a linear or near-linear relationship between angle/stroke/magnet strength/pressure, before reaching a maximum hardness and producing little to no further change.

**FIGURE 6:**
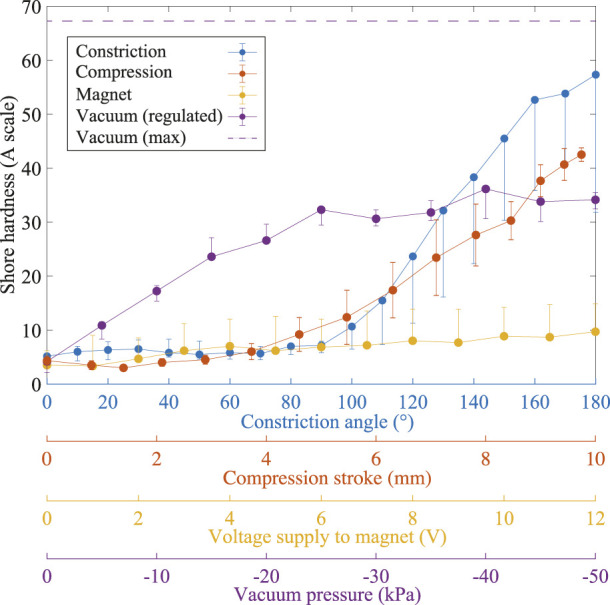
Average hardness measured in the centre of each touchpad using the constriction, compression, magnetic and vacuum actuation techniques. Also shown is the maximum hardness achieved using an unregulated vacuum pump (−100 kPa) which is the current state-of-the-art for particle jamming-based soft robots. Error bars show the minimum and maximum hardness measured elsewhere on the surface.

All of the above results are summarised in Figure 6.

### 4.2 Surface uniformity

**FIGURE 7:**
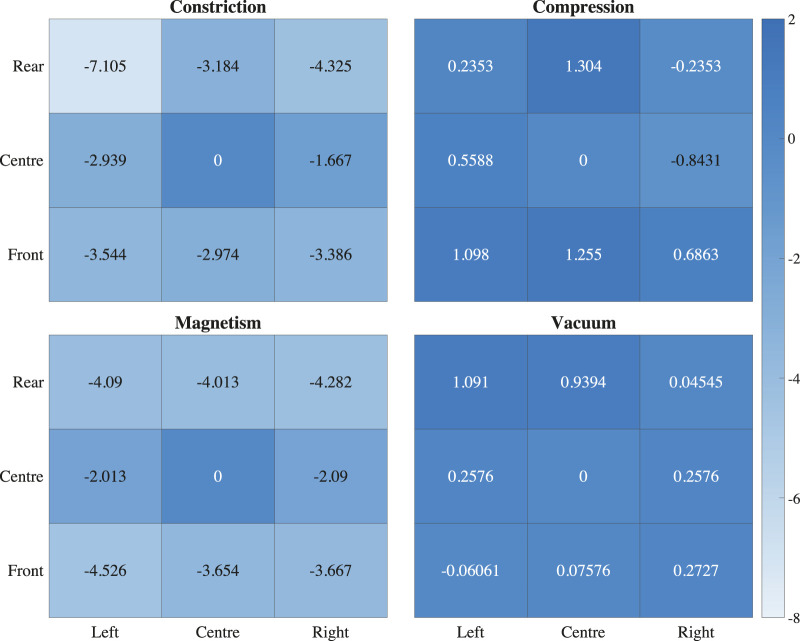
Average deviation in shore hardness across different regions of the surface of each prototype tactile display compared to its centre.

Surface uniformity is also an important consideration for tactile displays. The ideal tactile display will present a completely stable and deterministic sensation across its surface area. The hardness data taken from different regions of the touchpad indicates that none of the devices under test were able to produce completely uniform surface hardness. Averaging over different hardness steps, the vacuum powered and compression based devices showed the most uniform surface hardness, with average variances from the centre within ± 1.1A (vacuum) and ± 2.1A (compression). The magnetic and constrictive devices tended to become harder at the edges of the surface, which were found to be up to 4.5A harder than the centre, and never softer. The constrictive device was more variable still, with the edges measuring up to 7.1A harder than the centre of the touchpad. The changes in hardness over each tactile display surface are shown graphically as heatmaps in Figure 7.

### 4.3 Response time

An ideal tactile display (or any HCI device) will respond immediately to a command to change its output. This is impossible to achieve with engineered physical systems. However, a low response time is essential to create dynamic and interactive haptic experiences. The magnetic approach was the fastest to actuate as there is very little mechanical change that must take place to jam the particles, and in fact outperformed the time measurement setup, switching on in less than 0.05 s. The compression prototype was the next fastest, taking 0.270 s to move through its full 10 mm stroke and exhibit its maximum hardness change. The constriction prototype took 0.741 s to turn 180 °and fully jam the particle fluid, with the pneumatic device taking 2.16 s to drop to −50 kPa representing its hardest (controllable) state.

### 4.4 Surface geometry

Surface geometry is also an important consideration for tactile displays and physical medical simulators. Unintended deformations in the display could be mistaken for lumps, skin lesions or bony landmarks such as those in the knee and wrist. Whilst surface geometry was not measured during this study, changes in surface shape were observed and recorded.

**FIGURE 8:**
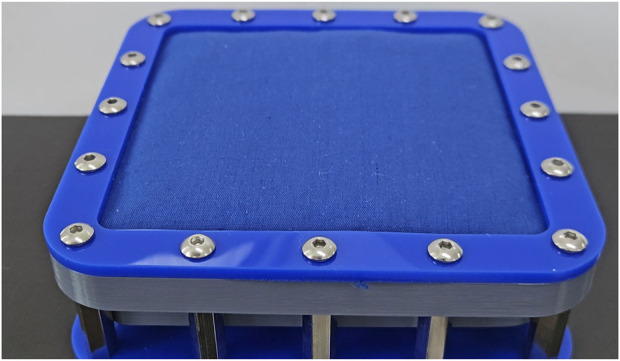
The “bulging” observed during mechanical particle jamming (compression prototype shown in it is hardest possible state).

The constriction and compression-based designs both experienced some swelling or bulging in their surface as slack in the fabric container was taken up by the twisting/compression motion of the device. This caused the surface to lift up moderately (<5 mm). This effect appeared uniform across each surface. After the initial slack was taken up, the surface geometry did not appear to change. This effect is illustrated in Figure 8.

**FIGURE 9:**
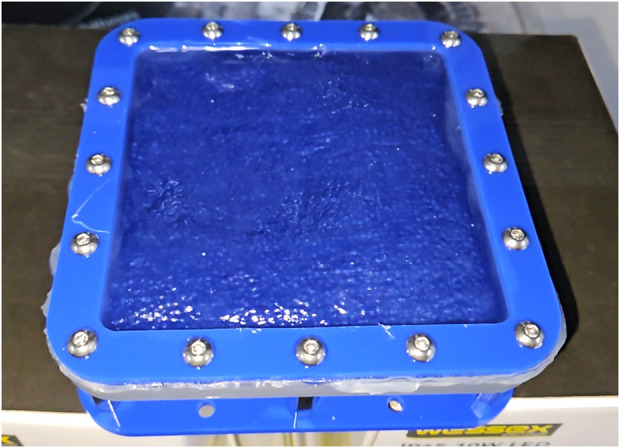
The “lumpy” touch surface observed after vacuum actuation (hardest possible state).

The vacuum-based approach caused the tactile display surface to contract and closely mould around the particles. This movement was very small (<2 mm) however the highly unstructured nature of the mechanism did cause some small bumps to appear on the surface, as well as the texture of the particles. The latter effect could be smoothed with a thicker surface. This is illustrated in Figure 9.

There was no noticeable change to surface geometry during magnetic actuation, though there may have been changes in the structure of the particle fluid within the display when the magnetic field was applied.

### 4.5 Non-functional characteristics

Aside from the above performance results, for a technology to be a viable candidate for use in medical training settings, it must also conform to certain (variable) requirements regarding its size, weight and power demand. Whilst weight and power consumption are highly dependent on the technology behind the implementation, the below discusses the size and cost of the various actuation methods presented above.

#### 4.5.1 Size

In order to generate robust and reliable comparisons from the performance data for each device, the size of each display and volume of each particle fluid was kept the same. There are, however, a number of other factors that affect the size and weight of each working system and these are extremely relevant to each technology’s viability for use in non-industrial devices, such as those that would be used to train medical students and junior doctors.

Each tactile display measured 100 mm × 100 mm × 30 mm. Adding a clamp and rigid frame around each increased the total size to 124 mm × 124 mm × 48.5 mm. The constriction device only required a high torque motor to be attached below the display, increasing its total size to 124 mm × 124 mm × 116.3 mm - an increase in height of approximately 70 mm. The eccentric cam mechanism used on the compression prototype allows the motor to be mounted more compactly, but the need to align with the centre of the piston does cause it to extend beyond the display area. This means an increase in the overall size to 124 mm × 138.7 mm × 83.4 mm. The magnet used in the magnetic prototype does not extend beyond the footprint of the tactile display and is relatively flat. This device measured 124 mm × 124 mm × 71.5 mm, including additional mounting hardware for the magnet. The vacuum powered device must be considered differently to the others as its power source does not need to be connected to it. Therefore, whilst the tactile display itself does not need to be any larger than the 124 mm × 124 mm × 48.5 mm unit described above, the additional pneumatic regulator (with associated controller and power supply) adds 159 mm × 97 mm × 47 mm, whilst the vacuum pump adds a further 290 mm × 170 mm × 120 mm. Air hoses are required between each of these subsystems, though they do not need to be co-located in one large unit.

#### 4.5.2 Cost

Cost is also an important factor for medical training aids. Medical schools may be distributed across a number of hospital sites, meaning that equipment and teaching aids must be duplicated on each site. Medical teaching aids or benchtop models also tend to be anatomical in design to resemble real patients or sections of human anatomy. Therefore, whatever system is used would potentially need to be applied to a range of benchtop models, each simulating a different part of the body. The cost of the system should therefore be kept to a minimum to be viable for widespread use in medical schools.

The constriction and compression devices both use identical electronic and mechanical hardware, arranged slightly differently. This consists of a geared DC motor, high speed microcontroller (Arduino Uno R4) for position control and motor driver module (L298N). Including custom manufactured plastic parts and the jamming fluid, these devices had a material cost of less than $30 each at end user prices. The magnetic version was more expensive. Although the magnet and control relay were cheaper than the motor, microcontroller and driver, the steel balls used as a ferromagnetic particle fluid cost $200. The pneumatic prototype was the most expensive, with the regulator costing $300, the vacuum pump adding a further $100, as well as approximately $50 for plastic and silicone, air hose, pneumatic connectors and a power supply and controller for the regulator. The total cost of each mechanism is therefore:

• Constriction: $30

• Compression: $30

• Magnetism: $230

• Vacuum: $450

The above results are summarised in Table 1.

**TABLE 1:** Summary of test and evaluation results.

Technology	Hardness range (shore units) (A)	Hardness uniformity (shore units) (A)	Response time (seconds)	Actuator volume (mm^3^)	Cost (approx USD)
Constriction	57	± 7.1	0.741	1,076,320	30
Compression	42.5	± 2.1	0.270	600,328	30
Magnetism	15	± 4.5	<0.05	353,648	230
Vacuum (regulated)	36	± 1.1	2.16	6,640,881	450

## 5 Discussion

The constriction approach demonstrated above showed the best controllable range of hardness change. Although the unregulated vacuum was able to produce a harder surface overall, this could not be repeated under controlled vacuum pressure. It was also the joint cheapest and lightest, as well as being the second smallest. It did however suffer from poorer surface uniformity results compared to the other techniques under test. It is implementation into wearable, handheld or shape changing devices may be complicated by the requirement for a rigid frame to extend to the top of the jamming pouch to constrain its rotation. It is response time is appropriate for use in an interactive haptic device.

The compression approach was found to be able to produce the second highest controlled hardness, after constriction. It was also found to be the second most uniform after the vacuum powered system, and the joint cheapest with the second smallest total volume. The linear piston driven mechanism is simpler to integrate with other devices than the constrictive approach, though not as simple as the magnetic design, or as flexible as the vacuum powered approach. It had the second fastest response time and it is eminently suitable for use in interactive haptic simulations.

The magnetic device performed relatively poorly in many respects. It achieved the smallest hardness change and exhibited the second worst hardness deviation across it is surface, though this was uniform and predictable. It was the second most expensive due to the large number of ferromagnetic steel balls, which also added considerable weight over the mechanical devices. The implementation is however extremely simple and easily reconfigurable, and may prove more effective with a smaller volume of particles.

Finally, whilst the vacuum powered device was able to produce the most significant change in hardness, this was not reproducible when driven by an industrial specification pressure regulator due to the pressure required to produce the effect. In this configuration, the controllable range of hardness change was the third lowest, though still within a suitable range for reproducing the feel of soft tissues, and had the most consistent surface uniformity. It was also the most controllable and the empirical hardness results demonstrated a very strong linear relationship between pressure and hardness. It was however the most expensive hardware setup tested due to the costs of the pressure regulator and vacuum pump, and also the heaviest and most bulky device to be tested. Lastly, this method had the slowest response time and would be unlikely to produce a good effect if used in an interactive haptic simulation.

These results show that, whilst the most effective hardness change is achieved using air pressure, this is dependent on the specification and performance capability of the individual components of the system. Electromechanical approaches can still achieve a comparable, and in some cases better, physical effect with a faster response time and at a much lower cost. All three devices were able to produce hardness changes in a range that would be useful for simulating soft tissues, such as liver and prostate, which have been found to occupy a region around 25-31A on the Shore scale by Estermann et al. (2020) and Baumgart et al. (2010), respectively, though in their current implementations may struggle to accurately represent very dense tissues like muscle, which has previously been recorded as having a hardness on the harder Shore C scale Kim et al. (2019).

### 5.1 Future work

Future work will use these technologies as a basis for a number of research directions. Particle jamming is an inherently flexible technology, and is well suited for modification to present hardness in combination with other haptic modalities, such as vibration to render pulses, temperature to indicate infection and abnormal blood flow, and texture to present skin lesions. Various approaches will be explored and tested, both in a lab and with users.

Further technical work will also explore how non-pneumatic actuation can be applied to other jamming techniques, including layer and fiber jamming, which have a range of applications in haptics, including in medical simulation and training.

Finally, future research aims to develop and test medical simulators based on the particle jamming approaches described above. The development of such simulators will explore approaches to creating localised areas of hardness and softness (for example, a hard lump within a soft abdomen) such as multi-cell arrays as used in Stanley et al. (2013), and encountered type soft tactile displays, enabled by recent advances in optical hand tracking.

User validation with both experienced clinicians and students at the Imperial College School of Medicine will evaluate these simulators’ realism and educational value for simulating physical pathologies, and training students in correct examination techniques. This is important for two reasons. Firstly, testing the devices with users will provide insight into the relationship between the quantitative hardness results presented above and perceived hardness through the hand during palpation. Prior evidence from Baumgart et al. (2010) suggests that they are closely linked. Secondly, user studies will provide data on the value of physical simulation in medical education contexts - the primary application area for these devices.

## Data Availability

The raw data supporting the conclusions of this article will be made available by the authors, without undue reservation.

## Footnotes

1 The Shore A scale is applicable to materials ranging from firm gels to engineering rubber and is routinely used in softness perception studies such as those described in Baumgart et al. (2010) and Zamani and Culbertson (2023).

## References

[B1] Aghassi D. Monoson T. Braverman I. (1995). Reproducible measurements to quantify cutaneous involvement in scleroderma. Archives Dermatology 131, 1160. 10.1001/archderm.1995.01690220066013 7574833

[B2] Al Maimani A. Roudaut A. (2017). Frozen suit: towarda changeable stiffness suit and its application for haptic games. Proc. 2017 CHI Conf. Hum. Factors Comput. Syst. 2017, 2440–2448. 10.1145/3025453.3025655

[B50] Baumgart L. A. Gerling G. J. Bass E. J. (2010). Psychophysical detection of inclusions with the bare finger amidst softness differentials Proc Symp Haptic Interface Virtual Env Teleoperator Syst 17–20. 10.1109/HAPTIC.2010.5444684 PMC2995217 21132051

[B3] Belyaev O. Herden H. Meier J. J. Muller C. A. Seelig M. H. Herzog T. (2010). Assessment of pancreatic hardness—surgeon versus durometer. J. Surg. Res. 158, 53–60. 10.1016/j.jss.2008.08.022 19394646

[B4] Biroli G. (2007). A new kind of phase transition? Nat. Phys. 3, 222–223. 10.1038/nphys580

[B5] Brancadoro M. Manti M. Tognarelli S. Cianchetti M. (2020). Fiber jamming transition as a stiffening mechanism for soft robotics. Soft Robot. 7, 663–674. 10.1089/soro.2019.0034 32250723

[B6] Brown E. Rodenberg N. Amend J. Mozeika A. Steltz E. Zakin M. R. (2010). Universal robotic gripper based on the jamming of granular material. Proc. Natl. Acad. Sci. U. S. A. 107, 18809–18814. 10.1073/pnas.1003250107

[B7] Brown J. Bello F. (2024). Design and characterisation of particle jamming-based variable stiffness displays using non-pneumatic actuators. IEEE Haptics Symposium, HAPTICS, Long Beach, CA, USA, 07-10 April, 2024 (IEEE) 379–384.

[B8] Brown J. Farkhatdinov I. (2021). “Shape-changing touch pad based on particle jamming and vibration,” in 2021 IEEE World Haptics Conference, WHC 2021, Montreal, QC, Canada, 06-09 July, 2021 (Institute of Electrical and Electronics Engineers Inc.), 337.

[B9] Brown J. P. Farkhatdinov I. (2020). “Soft haptic interface based on vibration and particle jamming,” in IEEE Haptics Symposium, HAPTICS, Washington DC, 2020-March (IEEE), 1–6.

[B10] Brown J. P. Farkhatdinov I. (2022). “Using audio recordings to characterise a soft haptic joystick,” in Lecture notes in computer science (including subseries lecture notes in artificial intelligence and lecture notes in bioinformatics). Editors Saitis C. Farkhatdinov I. Papetti S. (Germany: Springer Science and Business Media Deutschland GmbH), 102–111.

[B11] Chatzistergos P. E. Allan D. Chockalingam N. Naemi R. (2022). Shore hardness is a more representative measurement of bulk tissue biomechanics than of skin biomechanics. Med. Eng. and Phys. 105, 103816. 10.1016/j.medengphy.2022.103816 35781381

[B12] Draper M. H. Viirre E. S. Furness T. A. Gawron V. J. (2001). Effects of image scale and system time delay on simulator sickness within head-coupled virtual environments. Hum. Factors 43, 129–146. 10.1518/001872001775992552 11474759

[B13] Estermann S. J. Pahr D. H. Reisinger A. (2020). Quantifying tactile properties of liver tissue, silicone elastomers, and a 3D printed polymer for manufacturing realistic organ models. J. Mech. Behav. Biomed. Mater. 104, 103630. 10.1016/j.jmbbm.2020.103630 32174390

[B14] Follmer S. Leithinger D. Olwal A. Cheng N. Ishii H. (2012). Jamming user interfaces: programmable particle stiffness and sensing for malleable and shape-changing devices. UIST’12 - Proceedings of the 25th Annual ACM Symposium on User Interface Software and Technology. Pittsburgh, PA, USA, October 2012 (Association for Computing Machinery) 519–528.

[B15] Gaponov I. Popov D. Ryu J.-H. (2013). Twisted string actuation systems: a study of the mathematical model and a comparison of twisted strings. IEEE/ASME Trans. mechatronics 19, 1331–1342. 10.1109/tmech.2013.2280964

[B16] Hosseini M. Sengül A. Pane Y. De Schutter J. Bruyninck H. (2018). “Exoten-glove: a force-feedback haptic glove based on twisted string actuation system,” in 2018 27th IEEE International Symposium on Robot and Human Interactive Communication (RO-MAN), Nanjing, China, 08 November, 2018 (IEEE), 320–327.

[B17] Kim C.-Y. Kang J.-H. Park T.-S. (2019). Intra-rater reliability of the shore durometer in the assessment of upper trapezius muscle hardness. Res. J. Pharm. Technol. 12, 2461. 10.5958/0974-360X.2019.00412.8

[B18] Kissin E. Y. Schiller A. M. Gelbard R. B. Anderson J. J. Falanga V. Simms R. W. (2006). Durometry for the assessment of skin disease in systemic sclerosis. Arthritis Care and Res. 55, 603–609. 10.1002/art.22093 16874783

[B19] Kurihara Y. Koge M. Okazaki R. Kajimoto H. (2014). Large-area tactile display using vibration transmission of jammed particles. IEEE Haptics Symp. HAPTICS 1, 313–317. 10.1109/HAPTICS.2014.6775474

[B20] Li M. Ranzani T. Sareh S. Seneviratne L. D. Dasgupta P. Wurdemann H. A. (2014). Multi-fingered haptic palpation utilizing granular jamming stiffness feedback actuators smart mater. Struct 23, 95007. 10.1088/0964-1726/23/9/095007

[B21] Piaggesi A. Romanelli M. Schipani E. Campi F. Magliaro A. Baccetti F. (1999). Hardness of plantar skin in diabetic neuropathic feet. J. Diabetes its Complicat. 13, 129–134. 10.1016/S1056-8727(98)00022-1 10509872

[B22] Simon T. M. Smith R. T. Thomas B. H. (2014). “Wearable jamming mitten for virtual environment haptics,” in International Symposium on Wearable Computers, Washington, New York City, 13 September, 2014 (ACM) 67–70.

[B23] Skvortsova V. Nedelchev S. Brown J. Farkhatdinov I. Gaponov I. (2022). Design, characterisation and validation of a haptic interface based on twisted string actuation. Front. Robotics AI 9, 977367–977369. 10.3389/frobt.2022.977367 PMC9525208 36185974

[B24] Stanley A. A. Gwilliam J. C. Okamura A. M. (2013). “Haptic jamming: a deformable geometry, variable stiffness tactile display using pneumatics and particle jamming,” in 2013 World Haptics Conference, Daejeon, Korea (South), 14-17 April, 2013 (IEEE), 25–30. 10.1109/WHC.2013.6548379

[B25] Stanley A. A. Mayhew D. Irwin R. Okamura A. M. (2014). “Integration of a particle jamming tactile display with a cable-driven parallel robot,” in Haptics: neuroscience, devices, modeling, and applications. Editors Auvray M. Duriez C. (Berlin, Heidelberg: Springer), 258–265. 10.1007/978-3-662-44196-1_32

[B26] Stanley A. A. Okamura A. M. (2017). Deformable model-based methods for shape control of a haptic jamming surface. IEEE Trans. Vis. Comput. Graph. 23, 1029–1041. 10.1109/TVCG.2016.2525788 26863666

[B27] Tonna R. Chatzistergos P. E. Wyatt O. Chockalingam N. (2024). Reliability and validity of shore hardness in plantar soft tissue biomechanics. Sensors 24, 539. 10.3390/s24020539 38257632 PMC10818800

[B28] Ullrich S. Kuhlen T. (2012). Haptic palpation for medical simulation in virtual environments. Tech. Rep. 18, 617–625. 10.1109/tvcg.2012.46 22402689

[B29] Würtz T. May C. Holz B. Natale C. Palli G. Melchiorri C. (2010). “The twisted string actuation system: modeling and control,” in 2010 IEEE/ASME International Conference on Advanced Intelligent Mechatronics. Montreal, QC, Canada, 06-09 July, 2010 (IEEE) 1215–1220.

[B30] Zamani N. Culbertson H. (2023). Effects of physical hardness on the perception of rendered stiffness in an encountered-type haptic display. IEEE Trans. Haptics 16, 46–56. 10.1109/TOH.2022.3226182 37015574

[B31] Zubrycki I. Granosik G. (2017). Novel haptic device using jamming principle for providing kinaesthetic feedback in glove-based control interface. J. Intelligent Robotic Syst. Theory Appl. 85, 413–429. 10.1007/s10846-016-0392-6

